# Neoplastic and Non-neoplastic Acute Intracerebral Hemorrhage in CT Brain Scans: Machine Learning-Based Prediction Using Radiomic Image Features

**DOI:** 10.3389/fneur.2020.00285

**Published:** 2020-05-05

**Authors:** Jawed Nawabi, Helge Kniep, Reza Kabiri, Gabriel Broocks, Tobias D. Faizy, Christian Thaler, Gerhard Schön, Jens Fiehler, Uta Hanning

**Affiliations:** ^1^Department of Diagnostic and Interventional Neuroradiology, University Medical Center Hamburg-Eppendorf, Hamburg, Germany; ^2^Institute of Medical Biometry and Epidemiology, University Medical Center Hamburg-Eppendorf, Hamburg, Germany

**Keywords:** intracerebral hemorrhage, neoplastic hemorrhage, radiomics, machine learning, artificial intelligence

## Abstract

**Background:** Early differentiation of neoplastic and non-neoplastic intracerebral hemorrhage (ICH) can be difficult in initial radiological evaluation, especially for extensive ICHs. The aim of this study was to evaluate the potential of a machine learning-based prediction of etiology for acute ICHs based on quantitative radiomic image features extracted from initial non-contrast-enhanced computed tomography (NECT) brain scans.

**Methods:** The analysis included NECT brain scans from 77 patients with acute ICH (*n* = 50 non-neoplastic, *n* = 27 neoplastic). Radiomic features including shape, histogram, and texture markers were extracted from non-, wavelet-, and log-sigma-filtered images using regions of interest of ICH and perihematomal edema (PHE). Six thousand and ninety quantitative predictors were evaluated utilizing random forest algorithms with five-fold model-external cross-validation. Model stability was assessed through comparative analysis of 10 randomly drawn cross-validation sets. Classifier performance was compared with predictions of two radiologists employing the Matthews correlation coefficient (MCC).

**Results:** The receiver operating characteristic (ROC) area under the curve (AUC) of the test sets for predicting neoplastic vs. non-neoplastic ICHs was 0.89 [95% CI (0.70; 0.99); *P* < 0.001], and specificities and sensitivities reached >80%. Compared to the radiologists' predictions, the machine learning algorithm yielded equal or superior results for all evaluated metrics. The MCC of the proposed algorithm at its optimal operating point (0.69) was significantly higher than the MCC of the radiologist readers (0.54); *P* = 0.01.

**Conclusion:** Evaluating quantitative features of acute NECT images in a machine learning algorithm provided high discriminatory power in predicting non-neoplastic vs. neoplastic ICHs. Utilized in the clinical routine, the proposed approach could improve patient care at low risk and costs.

## Introduction

While quality and resolution in both computed tomography (CT) and magnetic resonance imaging (MRI) technology has greatly increased in the past decades, the interpretation of images remains largely descriptive, subjective, and non-quantitative (1). With the expansion of computational power and information content in clinical imaging data, novel machine learning-based algorithms increasingly contribute to patient-specific diagnosis and treatment, especially in neuro-oncology (2, 3).

About 10% of intracerebral neoplastic lesions initially present as spontaneous hemorrhagic stroke (4). Acute non-contrast-enhanced computed tomography (NECT) imaging is the preferred screening method when intracerebral hemorrhage (ICH) is suspected; however, follow-up imaging is required for final diagnosis (4, 5). Interpretive challenges emerge from intra-hemorrhage and spatial heterogeneity as well as from the wide variety of different encompassing entities (6). Hence, initial radiological evaluation may be unreliable (4, 7). A recent pooled analysis identified 18 reported cases of glioblastoma-induced hemorrhage that were misdiagnosed as hypertensive ICHs, leading to significant diagnostic delays in two-thirds of the cases (7). Frequently, time-consuming and often negative neurovascular workup is being performed additionally (7). In these cases, extraction of quantitative radiomic image features and evaluation of these data in automated machine learning approaches might offer additional information for discriminating neoplastic and non-neoplastic ICHs. Facilitating early and sensitive detection of neoplastic hemorrhage, such an approach could optimize diagnostic workup, reduce misclassifications and delayed final diagnosis, and hence improve patient care at low risk and cost in the clinical routine.

Radiomic analysis is built on the hypothesis that imaging data reflect the underlying morphology and dynamics of smaller-scale biologic phenomena (8, 9). In this context, two important imaging markers of ICH have been described: firstly, the presence and extent of perihematomal edema (PHE), and secondly, the dynamics of hemorrhage attenuation (4, 10). However, radiomic analysis aims to capture also image information not assessable by human eyes, such as texture metrics or the evaluation of filtered images.

We hypothesized that quantitative radiomic image features extracted from NECT brain scans can be used to differentiate neoplastic and non-neoplastic ICHs. To test and evaluate this hypothesis, we employed a previously published and established radiomics machine learning approach on NECT brain scans of patients presenting with acute ICH of unknown etiology (3, 11). Furthermore, we evaluated the predictive performance of the proposed algorithm in comparison to conventional visual assessments of two radiologist readers.

## Methods

This single-center retrospective study was approved by the ethics committee (Ethik-Kommission der Ärztekammer Hamburg, WF-054/19), and written informed consent was waived according to paragraph 9 section 2 of the Hamburg federal state legislation and paragraph 15 section 1 of the medical association's professional code of conduct in Hamburg. All study protocols and procedures were conducted in accordance with the Declaration of Helsinki. The data that support the findings of this study are available, upon reasonable request from the corresponding author, if in accordance with the institution's data security regulations.

**Figure 1:**
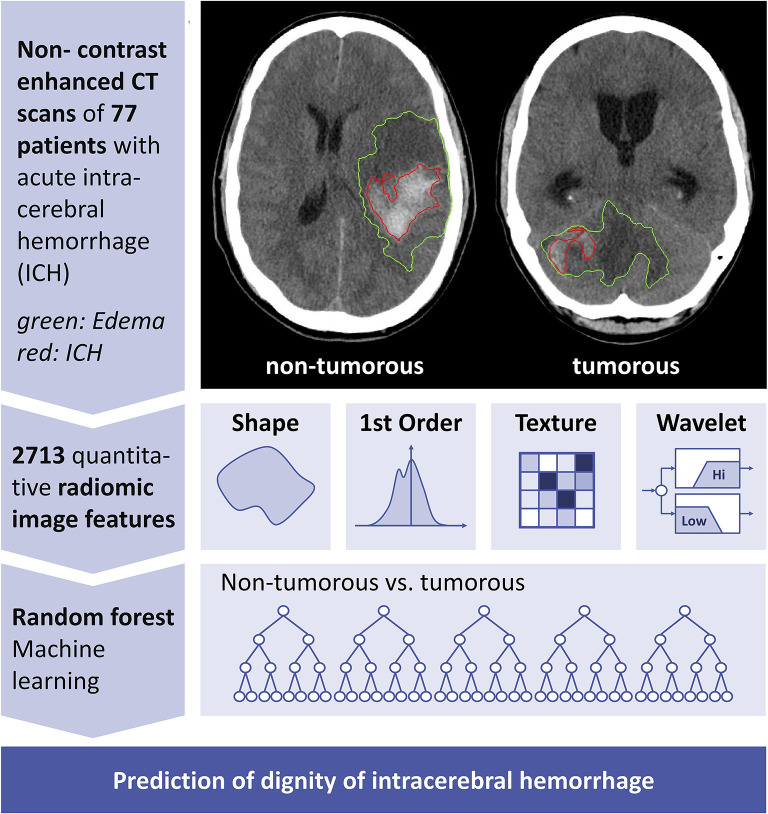
Conceptual overview of proposed neoplastic intracerebral hemorrhage prediction. Conceptual overview of the proposed machine learning approach showing the major processing steps: CT-based image acquisition and segmentation, feature extraction (*n* = 2,713), and statistical learning (random forest algorithm). NECT, non-contrast-enhanced computed tomography; ICH, intracerebral hemorrhage; PHE, perihematomal edema.

A graphical flow chart of the proposed machine learning-based prediction of the ICH etiology is shown in Figure 1, its components are detailed in the following.

### Patients

We retrospectively reviewed the database of our center for patients with acute ICH in whom NECT imaging was performed from January 2010 through December 2017. Patients were consecutively included according to inclusion following criteria: (1) acute, non-traumatic single subcortical, or lobar ICH, (2) NECT imaging within 72 h, (3) MRI follow-up imaging confirming cause of acute ICH, and (4) documented time of symptom onset. In cases of suspected vascular malformation, additional digital subtraction angiography (DSA) was performed. Out of 560 systematically reviewed patients, 136 patients met the inclusion criteria. Fifty-nine patients were retrospectively excluded from the study for the following reasons: intraventricular hemorrhage or subarachnoid hemorrhage (SAH)–predominant cases (*n* = 43); multiple hemorrhagic lesions (*n* = 9); cerebral venous thrombosis as cause of ICH (*n* = 5); and aneurysm-associated ICH (*n* = 2). Extracted clinical patient data comprised patient age and patient sex. Seventy-seven patients (*n* = 27 with neoplastic, *n* = 50 with non-neoplastic ICH) remained in the final study population (Table 1). Median age of patients with neoplastic ICH was 71 years [inter-quartile range (IQR): 63–75], 40.7% females; median age of patients with non-neoplastic ICH was 72 years (IQR: 54.8–79.0), 56% females. Among the 77 study patients, 11 had primary ICHs (*n* = 11), 12 patients had an underlying vascular malformation or a cavernoma (AVM, *n* = 5; cavernoma, *n* = 7); 6 patients had an underlying amyloid angiopathy (*n* = 6); 21 patients had an unclear but neither neoplastic nor vascular pathology (*n* = 21), 21 patients had underlying brain metastasis (*n* = 21), and 6 patients had primary brain tumors (*n* = 6). All diagnoses were confirmed by follow-up MRI. Study patients were dichotomized for the binary outcome neoplastic vs. non-neoplastic ICH. Age, sex, time interval from symptom onset to NECT, and localization of ICH were not significantly different (*P* > 0.05; Table 1).

**Table 1:** Demographic data of study population.

**Baseline characteristics**	**Non-neoplastic ICH (*n* = 50)**	**Neoplastic ICH (*n* = 27)**	***P*-value**
Age (years), mean (mean ± SD)	72 (54; 79)	71 (62; 75)	0.70
Sex female, *n* (%)	28 (56.0)	11 (40.7)	0.20
Time onset to imaging (h), median (IQR)	8.25 (2.88; 24)	21.0 (4.0; 54.0)	0.06
Localization supratentorial, *n* (%)	46 (92.0)	23 (85.2)	0.35
Density (HU), mean (mean ± SD)	54.6 (54.8; 79.0)	48.0 (40.00; 54.6)	0.001
Total hemorrhage volume (cm^3^), median (IQR)	35.5 (16.7; 72.4)	47.2 (47.2; 108.2)	0.13
ICH volume (cm^3^), median (IQR)	15.4 (63.8; 36.0)	13.2 (8.7; 32.1)	0.54
PHE volume (cm^3^), median (IQR)	13.6 (7.7; 34.9)	38.2 (11.4; 80.5)	0.007

*Continuous variables are represented as mean ± standard deviation (SD) and categorical variables as number (n), and percentages (%). IQR, inter-quartile range; ICH, intracerebral hemorrhage; PHE, perihematomal edema; HU, Hounsfield units*.

### Image Acquisition

All patients received stroke imaging protocols at admission with NECT performed in equal order on 256 dual slice scanners (Philips iCT 256). NECT brain images were obtained from the vertex to the skull base (120 kV, 280–320 mA, 4.0 mm slice thickness, <0.6 mm in plane resolution). Additional CT angiography (CTA) was partially performed when atypical ICH was suspected. CT perfusion (CTP) was omitted. All NECT data sets were inspected for quality and excluded in case of severe motion artifacts as described in the section above.

### Segmentation of Intracerebral Hemorrhage and Perihematomal Edema

ICH and PHE were segmented semi-automatically by two MDs (UH: 8 years clinical experience in diagnostic neuroradiology in an academic full-service hospital, research with focus on clinical applications of image processing and predictive modeling; JN: 2 years clinical experience in diagnostic neuroradiology in an academic full-service hospital) on the basis of the original NECT images. Both readers were blinded to all clinical information. Regions of interest (ROIs) were delineated using Analyze 11.0 Software (Biomedical Imaging Resource, Mayo Clinic, Rochester, MN). Consensus ROIs were derived based on overlapping segmentations of both readers.

### Machine Learning Approach

Machine learning-based classification was performed using random forest algorithms [Python scikit-learn environment v0.18.1 (12)]. Random forest classifiers were shown to have a comparably low tendency to overfit (13) and allow classification tasks also for data sets with a large number of heterogeneous predictors. Based on stability analysis of the total model out-of-bag error, the number of trees was set to 500, and the number of features per node was set to the square root of the total number of features (13).

### Model Validation

Model validation was conducted using five-fold cross-validation with independent training and validation sets in a model-external approach (14). Model stability was examined through comparative analysis of 10 randomly permuted cross-validation sets.

### Feature Extraction

Extracted radiomic features were defined according to the PyRadiomics Python package v2.1.0 (11), ROIs were resampled to 1 × 1 × 1 mm isotropic resolution using sitk BSpline interpolators. Extracted features comprised 252 first-order features (thereof 18 based on unfiltered images, 144 based on wavelet decompositions, 90 based on log-sigma Laplacian of Gaussian filters), 902 texture features (thereof 68 based on unfiltered images, 544 based on wavelet decompositions, 290 based on log-sigma Laplacian of Gaussian filters), and 14 shape features. In total, 1,218 quantitative image features were extracted from the ICH, PHE, and ICH plus PHE ROIs. Furthermore, feature ratios of ICH/PHE and ICH/(PHE plus ICH) were calculated, resulting in a total of 6,090 extracted quantitative image features.

In brief, shape features were extracted from the hemorrhage and edema ROIs and do not depend on gray level distributions of the image. Shape features include descriptors of the three-dimensional size and shape of the ROI, e.g., volume, surface area, diameter, and sphericity. First-order and texture features were derived from the original images, from wavelet filtered images (high and low passes in three different directions), and from log-sigma-filtered images [log-sigma function at different sizes (1–5, 1 mm increment]. First-order statistics describe the distribution of voxel intensities within the image region defined by the ROI through basic metrics, e.g., mean, median, percentiles, and kurtosis. Texture features quantify the distribution of gray levels in an image with regard to, e.g., the size and position of zones of equal gray levels. The gray level co-occurrence matrix (GLCM) represents the number of times specific combination of gray levels occur in two pixels of an image that are separated by a specific distance. The gray level size zone matrix (GLSZM) quantifies specific gray level zones in an image. The gray level run length matrix (GLRLM) quantifies gray level runs that are defined as the length of consecutive pixels that have the same gray level value. The neighboring gray tone difference matrix (NGTDM) quantifies the difference between a gray value and the average gray value of its neighbors. The gray level dependence matrix (GLDM) quantifies gray level dependencies in an image. A gray level dependency is defined as the number of connected voxels within a specific distance that are dependent on the center voxel.

### Feature Selection

Selection of features with the highest predictive value was performed separately for each training data set considering Gini impurity measures (15). Feature sets with outliers greater than six standard deviations (SDs) were excluded from the analysis. For final model training and validation, we employed the 100 most important features of each set.

### Radiologist Reading

Two MDs (UH, JN) predicted the dignity of ICHs based on the acute NECT images. For each ICH, the readers rated “neoplastic” or “non-neoplastic.” Both readers were blinded to the ground truth, the classifier prediction, and the other reader's prediction.

### Statistics

The shown receiver operating characteristic (ROC) curve was calculated based on means of all cross-validation sets. For each set, classifiers were trained and tested on the set's unique training and validation samples employing the 100 most important features of the respective training data. Hence, mean ROC curves can be considered as unbiased estimates of general model classification performance. Statistical significance of the mean area under the curve (AUC) was assumed if *P* < 0.05 for all cross-validation sets. Model prediction instability was derived from the SD of ROC curves. *P*-values were calculated according to Mann–Whitney/Wilcoxon U statistics using the verification R-package v1.42 (16). Confidence intervals (CIs) for sensitivities and specificities were bootstrapped (2,000 replicates) using pROC v1.10 (17) and qwraps2 v0.3.0 R-packages. Statistical significance of differences in specificities was evaluated with McNemar test statistics (DTComPair v1.0.3 R-package). Total classification performance of radiologist readers and the machine learning classifier was compared using the Matthews correlation coefficient (MCC) (18). MCC integrates all fields of the confusion matrix and is generally considered as a favorable metric for unbiased comparisons of binary classifiers (19). Further, MCC evaluates balance ratios of the four confusion matrix categories (true positives, true negatives, false positives, false negatives) and allows comparison of classifiers also for unbalanced data sets (20–22). With *TP*: true positives, *TN*: true negatives, *FP*: false positives, and *FN*: false negatives, MCC is defined as:

$$\begin{array}{r} MCC=\frac{TP\;x\;TN-FP\;x\;FN\;}{\sqrt{\left(TP+FP\right)\left(TP+FN\right)\left(TN+FP\right)\left(TN+FN\right)}} \end{array}$$

Statistical significances of differences in MCC were calculated using the “psych” v1.8.12 R-package.

## Results

Our analysis includes NECT images of 77 patients with acute ICH, thereof 50 with non-neoplastic and 27 with neoplastic cause defined by final diagnosis in follow-up MRI.

### Classifier Performance

**Figure 2:**
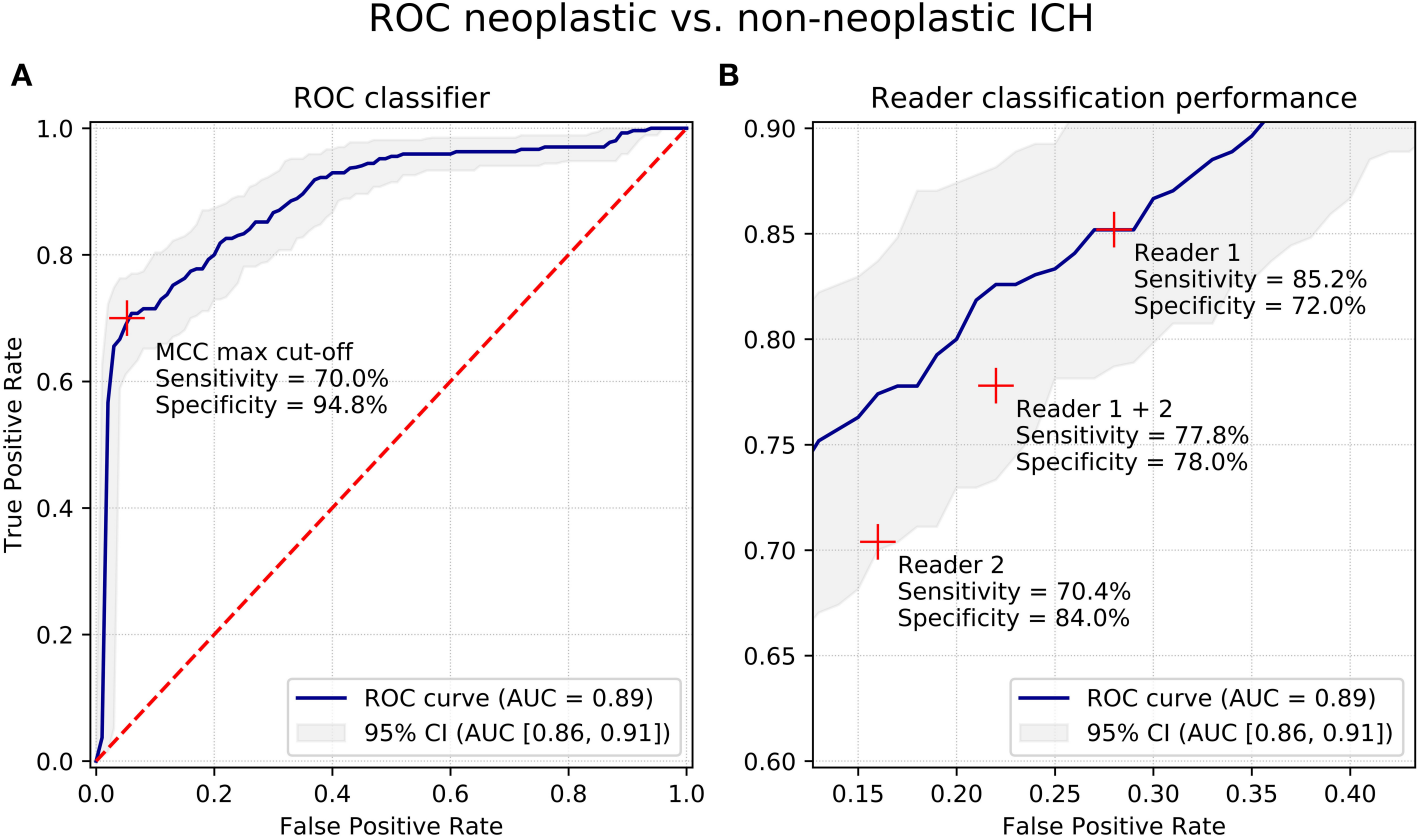
Receiver-Operating-Characteristics curves for differentiation of neoplastic and non-neoplastic ICHs. **(A)** Receiver-Operating-Characteristics (ROC) curves for differentiation of neoplastic and non-neoplastic ICHs of the proposed machine learning classifier based on quantitative radiomic image features. **(B)** Cut-out of panel **(A)** showing classification results of human reader 1 and 2. Blue line shows ROC curve, grey area shows 95% confidence interval (CI). Red crosses show cut-off points/prediction performance. AUC, area under the curve; CI, confidence interval; ROC, Receiver-Operating-Characteristics; ICH, intracerebral hemorrhage; MCC, Matthews correlation coefficient.

ROC AUC of the validation sets for predicting the dignity of ICH was 0.89 [95% CI: (0.70; 0.99); SD: 0.013]; all *P* < 0.01. Depending on selected cutoff values, the classifier yielded specificities and sensitivities of >80% (Figure 2A). The highest MCC measures of 0.69 were calculated at 70% sensitivity and 95% specificity with a Youden index of 0.65 and accuracy of 86% (Table 2).

**Table 2:**
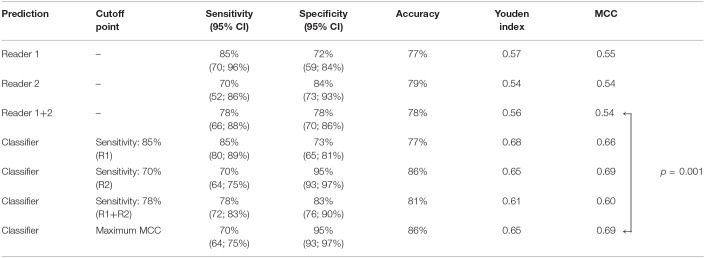
Classification performance metrics of radiologist readers and machine learning classifier.

*Classifier metrics are shown at cutoff points according to radiologist readers' sensitivities and at the classifiers' optimal operating point. MCC at the classifier's optimal operating point (0.69) is significantly higher compared to the combined result of readers 1 and 2 (0.54); P = 0.01. MCC, Matthews correlation coefficient; CI, confidence interval*.

### Feature Importance

**Figure 3:**
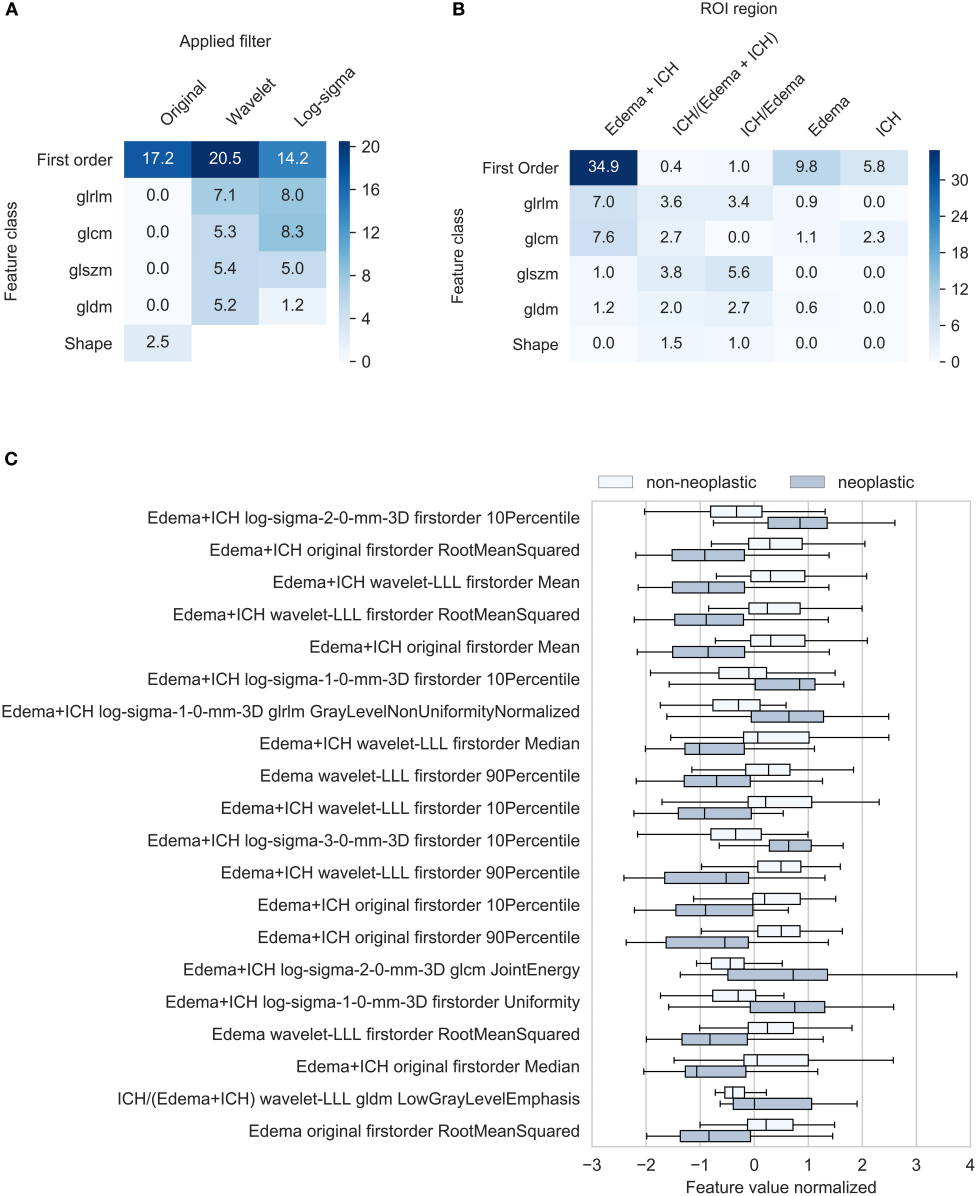
Characterization of most important features. Feature importance contribution of 100 most important features in % **(A)** By applied filter and feature class **(B)** by region and feature class. Texture feature class includes gray level size zone matrix, gray level dependence matrix, gray level run length matrix, and gray level size zone. **(C)** Radiomic feature signatures of neoplastic and non-neoplastic intracerebral hemorrhage. Box-plots show normalized means of the 20 most important image features. All mean feature values significantly different between neoplastic and non-neoplastic ICHs (*P* < 0.05). ROI, region of interest; ICH, intracerebral hemorrhage; PHE, perihematomal edema; gldm, gray level dependence matrix; H, high-pass wavelet decomposition; L, low-pass wavelet decomposition; glnu-norm, gray level non-uniformity normalized; RMS, root mean squared.

The top-100 features with the highest predictive power were mainly derived from ROIs comprising both PHE and ICH segmentations (52% of total predictive power). The lowest predictive value was calculated for ICH segmentations alone (8%) (Figure 3A). Regarding feature classes, fist-order histogram-based measures and texture features ranked highest with 52 and 46% of total predictive power, and shape-based features only contributed 2.5%. Filter-based extractions significantly increased predictive power: Wavelet and log-sigma-filtered images contributed 44 and 37%; unfiltered images contributed only 20% to total predictive power (Figure 3B). Of the 100 most important feature values, 86 were significantly different for neoplastic and non-neoplastic ICHs (*P* < 0.05). Normalized feature value box plots of the 10 most important predictors demonstrate differences in feature expressions for non-neoplastic and neoplastic ICHs and show typical radiomic signatures of the entities (Figure 3C). The most important feature comprises both ROIs, PHE, and ICH, and measures the 10th percentile of a 2 mm log-sigma-filtered image. Features #2 to #5 are first-order density metrics extracted from original and wavelet low-pass filtered (LLL) images.

### Radiologist Reading

Reader 1 predicted the dignity of ICHs with a sensitivity of 85% and a specificity of 72%; accuracy was 77%, Youden index was 0.57, and MCC was 0.55. Reader 2 achieved 70% sensitivity at 84% specificity with accuracy of 79%, Youden index of 0.54, and MCC of 0.54 (Figure 2B, Table 2).

### Comparison of Classifier and Radiologist Reader Prediction Performance

Comparative analysis of specificities at the reader's sensitivity set points suggests that classification performance of the machine learning algorithms was equal or superior for all evaluated metrics. Whereas reader 1 achieved classification results equivalent to the proposed algorithm, the metrics of reader 2 were lower, with specificity at −11% (84 vs. 95%, *P* = 0.06) and MCC at −0.15 (0.54 vs. 0.69, *P* = 0.08). When comparing the combined human rating results (reader 1 and reader 2) with the classifier's predictions at its optimal operating point, MCC of the proposed algorithm (0.69) was significantly higher than MCC of the radiologist readers (0.54); *P* = 0.01 (Table 2).

## Discussion

The main findings of our study are, firstly, that the proposed machine learning approach employing quantitative image features derived from NECT scans provides high discriminatory accuracy in predicting neoplastic ICHs. Secondly, depending on the classifier operating point, the proposed algorithm reaches significantly higher MCC metrics compared to visual ratings.

The proposed classifier yielded an AUC of 0.89 for the prediction of neoplastic ICHs with sensitivities and specificities reaching >80% depending on the cutoff value. Narrow CIs and low SDs of ROC curves suggest high stability of predictive performance. Whereas visual ratings of an 8-years-experienced senior neuroradiologist (reader 1) yielded similar metrics, results of the less experienced reader 2 were inferior, with a −11% loss in specificity (*P* = 0.06). Overall, MCC, a widely accepted metric for comparing binary classifiers, was significantly higher for the machine learning algorithm, with 0.69 vs. 0.54 for visual ratings of readers 1 and 2 (*P* = 0.01) (19). Hence, utilized as a supportive decision tool in clinical practice, the proposed algorithm improved and facilitated initial triaging, diagnostic workup, and precision of final diagnosis in patients presenting with acute ICHs. Also, the utilization of the tool for training and quality control especially for inexperienced residents is an interesting aspect, as MCC was different between the resident and the experienced neuroradiologist.

Although numerous interrelations between quantitative image features and clinical diagnoses have been demonstrated, radiomic analyses are still lacking wide clinical acceptance (2). In particular, the missing link between quantitative metrics, traditional imaging features, and the underlying biology has been a major point of criticism (2). To address these concerns, we evaluated the employed quantitative predictors with respect to their interpretation in visual assessments and established ties to traditional semantic imaging features. It is widely accepted that tumors and metastases are surrounded by an extensive PHE prior to a bleeding event. Preliminary studies underline the CT-based diagnostic importance of this pathophysiological process, as recently published (23). In line with this, Choi et al. (4) have described that a reduced hematoma attenuation in ICH can differentiate neoplastic from non-neoplastic lesions with high diagnostic accuracy. Accordingly, our analysis of the 100 most important features demonstrates that intensity distribution-based predictors (first-order histogram) contribute 51.9% of the cumulated feature importance (Figure 3A). Corresponding to classic semantic image readings, our by-region assessment shows that image features extracted from the entire lesion (ICH and PHE) yield the highest contribution (52%) to predictive performance (Figure 3B). However, our analysis also proves that the NECT imaging information is much richer: With a 45.6% share in cumulated importance, texture features play a similar important role as classic first-order predictors (Figure 3A). Furthermore, differentiation of features by applied filter demonstrates that wavelet and log-sigma-filtered images with a contribution of 44 and 37%, respectively, yield superior importance compared to non-filtered images, with a share of 19% (Figure 3A). Figure 3C shows box plots of normalized feature values of the 10 most important predictors for neoplastic and non-neoplastic ICHs. The graph demonstrates that the 10th percentile of log-sigma (2 mm) filtered images is the metric with the highest predictive power. This suggests that neoplastic ICHs express significantly sharper density edges compared to non-neoplastic ICHs. Features #2 to #5 are intensity measures extracted from original and from wavelet low-pass filtered images. In line with clinical studies proposing hematoma density as a diagnostic marker for neoplastic ICH on CT (4), these metrics suggest that neoplastic lesions are hypodense compared to non-neoplastic ICHs.

To our knowledge, this is the first study that investigates the use of quantitative radiomic image features extracted from NECT scans to differentiate neoplastic and non-neoplastic ICHs. The proposed method integrates the merits from quantitative radiomic features and machine learning algorithms and relates the employed predictors to traditional radiographic imaging findings. Unlike our study, existing radiomics-based analyses regarding CT imaging have mainly focused so far on prompt ICH diagnosis and automated volume quantification (24, 25).

Our study had general limitations typically associated with quantitative radiomics-based image analysis and classification (3, 8, 26, 27). These limitations include differences in image acquisition techniques, under- or overfitting of machine learning algorithms, and potential misclassifications in the ground truth definitions. All of these limitations could bias classification and may lead to less generalizable results. Furthermore, we observed study-specific limitations: First, we only included a limited number of patients in a retrospective analysis. An expansion of sample size in a prospective study design would certainly contribute to further improving generalizability of results. Small sample sizes are a general concern for radiomics analysis and are due to the limited availability of standardized multi-center databases. However, results of our model stability analysis suggest sufficient robustness for assessing general feasibility and limitations of the proposed algorithm. Second, the manual definition of ROIs still implies a certain degree of observer-dependence within the machine learning process. To minimize its influence, we employed consensus segmentations from two independent readers and applied a semi-automated delineation segmentation method that was shown to have favorable inter- and intra-observer reliability (10). Noteworthily, variabilities are lower in automatic vs. semi-automatic vs. manual delineation; however, semi-automatic delineation was mandatory in our case (28–31). Further, it was shown that radiomic features are comparably stable with regard to variations in segmentations (30, 32). Third, the underlying NECT images of our analysis were acquired with the same scanner at the same hospital. This might reduce generalizability of results. However, due to standardized and calibrated quantitative imaging parameters and signal intensity processing of CT scanners, we assume neglectable bias on classifier performance in a generalized setting. Lastly, the hematoma density difference between neoplastic and non-neoplastic ICHs can be discussed critically, as symptom onset to imaging time differed by trend between the two categories. As ICH density decreases over time, this might have biased results. However, the difference in onset to imaging times was statistically not significant and in line with current literature (4).

From our results, we conclude that the additional imaging information extracted through texture analysis and filtering as well as the standardized and fully automated machine learning algorithm is the main factor determining the observed high prediction performance and stability. As this information is not assessable by human eyes, the proposed approach can be used as supportive tool to improve the radiologist's diagnostic decision. Through facilitating efficient triage, reducing initial misclassifications, and preventing delayed diagnosis, the proposed algorithm could improve patient care in the daily clinical routine at low risk and costs.

## Data Availability Statement

The data that support the findings of this study are available, upon reasonable request from the corresponding author, if in accordance with the institution's data security regulations.

## Ethics Statement

The studies involving human participants were reviewed and approved by Ethik-Kommission der Ärztekammer Hamburg, WF-054/19. The Ethics Committee waived the requirement of written informed consent for participation.

## Author Contributions

JN contributed to the study design, the patient data collection, took a lead in writing the manuscript, conceived, and planned the experiments. HK contributed to the image processing, image analysis, statistical analysis, data analysis, drafting the manuscript, and revising it critically. RK contributed to the acquisition of data, drafting the manuscript, and revising it critically. GB, TF, and CT contributed to the data analysis, drafting the manuscript, and revising it critically. GS contributed to the statistical analysis, data analysis, drafting the manuscript, and revising it critically. JF contributed to the study design, data analysis, drafting the manuscript, and revising it critically. UH contributed to the study design, acquisition of data, image analysis, data analysis, drafting the manuscript, and revising it critically.

## Conflict of Interest

JF: Research support: German Ministry of Science and Education (BMBF), German Ministry of Economy and Innovation (BMWi), German Research Foundation (DFG), European Union (EU), Hamburgische Investitions- und Förderbank (IFB), Medtronic, Microvention, Philips, Stryker. Consultant for: Acandis, Boehringer Ingelheim, Cerenovus, Covidien, Medtronic, Microvention, Penumbra, Stryker. The remaining authors declare that the research was conducted in the absence of any commercial or financial relationships that could be construed as a potential conflict of interest.
