# Corrigendum: Neoplastic and Non-neoplastic Acute Intracerebral Hemorrhage in CT Brain Scans: Machine Learning-Based Prediction Using Radiomic Image Features

**DOI:** 10.3389/fneur.2021.687610

**Published:** 2021-05-21

**Authors:** Jawed Nawabi, Helge Kniep, Reza Kabiri, Gabriel Broocks, Tobias D. Faizy, Christian Thaler, Gerhard Schön, Jens Fiehler, Uta Hanning

**Affiliations:** ^1^Department of Diagnostic and Interventional Neuroradiology, University Medical Center Hamburg-Eppendorf, Hamburg, Germany; ^2^Institute of Medical Biometry and Epidemiology, University Medical Center Hamburg-Eppendorf, Hamburg, Germany

**Keywords:** intracerebral hemorrhage, neoplastic hemorrhage, radiomics, machine learning, artificial intelligence

In the original article, there was an error. The article erroneously states that “The data that support the findings of this study are available from the corresponding author upon reasonable request.” Unfortunately, we are unable to provide the full raw data scan set due to the recent implementation of stricter data security regulations by our institution.

A correction has been made to *Methods, Paragraph 1*. The corrected paragraph is shown below.

This single-center retrospective study was approved by the ethics committee (Ethik-Kommission der Ärztekammer Hamburg, WF-054/19), and written informed consent was waived according to paragraph 9 section 2 of the Hamburg federal state legislation and paragraph 15 section 1 of the medical association's professional code of conduct in Hamburg. All study protocols and procedures were conducted in accordance with the Declaration of Helsinki. The data that support the findings of this study are available, upon reasonable request from the corresponding author, if in accordance with the institution's data security regulations.

The Data Availability Statement has also been updated, as shown below.

## Data Availability Statement

The data that support the findings of this study are available, upon reasonable request from the corresponding author, if in accordance with the institution's data security regulations.

The authors apologize for this error and state that this does not change the scientific conclusions of the article in any way. The original article has been updated.

